# The Institutional Library

**Published:** 1914-01-10

**Authors:** 


### THE INSTITUTIONAL LIBRARY.

### The Sanatorium Routine.

Sanatoria for the Tuberculous. By Dr. F. R. Walters. (George Allen and Co. Demy 8vo. Pp. 436. 12s. 6d. net.)

The present volume is the fourth edition of Dr. Walters' work, which first appeared in 1899. The great changes and advances that have been made since the last edition was published in 1905 have necessitated the entire re-writing of this excellent treatise. The labour entailed in bringing a work of this nature up to date is immense, and the author is to be congratulated on the successful result. Owing to the temporary arrangements that have been made in many places the descriptions are naturally in some cases incomplete. It is interesting to note that, notwithstanding the exceptional activity displayed in England owing to the National Insurance Act, the countries of Europe and the United States have not been less active in providing institutions. It is noteworthy that in Germany very much greater provision has been made for children than at present exists in this country. In addition to fifteen sanatoria with 861 beds there are "about ninety convalescent homes (with nearly 8,000 beds) for children threatened with tuberculosis, or scrofulous, or merely run down in health." This is certainly an example which we should do well to follow. Unfortunately, it cannot be said that Germany has made anything like adequate provision for advanced cases, an error into which our own authorities seem likely to fall. The description of the arrangements made by New York City, which are wonderfully complete, should stimulate the London and Middlesex County Councils to action and emulation. America has certainly something to show us in this respect, and, though the conditions in New York are not as complicated as in London, we are very far behind them in the treatment of tuberculosis. The present volume is divided into two parts, the first of which deals with the principles and methods of sanatorium treatment and is more complete in this respect than previous editions. Sites and climates, the buildings, construction, staff, and management are all dealt with concisely. The chapter on climates is particularly valuable, and should tend to check the giving of unwise advice on this question and prevent patients from being indiscriminately ordered away from their own countries, in many cases to die among strangers. The principles of treatment, the selection and classification of patients, and the routine in a sanatorium are considered, and some suggestions made on the difficult subject of classification of results.

Part II. is devoted to a description of the various sanatoria in England and the Colonies, also the United States and the European Continent. It is not a mere classification of institutions, but consists of a necessarily short description of the site, soil, and buildings with the heating and lighting arrangements. The method of approach is also indicated. In many cases the cost of construction and the weekly or annual cost per bed is shown. The terms of admittance are stated in the majority of cases, and this is a feature which, if extended to all cases, would certainly add to the practical value of the book. The descriptions are often accompanied by excellent illustrations and plans. This excellent and instructive handbook can be thoroughly recommended, and should certainly be read by all sanatorium officials.

Gould and Pyle's Pocket Cyclopædia of Medicine and Surgery. Second Edition. By R. J. E. Scott, M.D. (London: H. K. Lewis. Pp. 732. Price 5s. net.)

The first edition of this pocket reference book was published twelve years ago, and it is no surprise to learn that for its reissue extensive revision has been necessary. Nevertheless the student of medicine, for whose use as a memory refresher the work would seem to be designed, must not take everything he finds in it for gospel, or he will get into trouble with his examiners. The arrangement of the subjects touched on is alphabetical, and a brief research through the A's is enough to reveal errors and deficiencies. Amenorrhœa and anæsthesia are instances in point. The information on these topics is not only imperfect and liable to misinterpretation, but is in some respects grossly wrong. Proportion, too, is often noticeably lacking; aconite, for instance, is allotted more space than alcohol and albuminuria put together. It would not be fair, however, to give the impression that weak spots exceed strong ones. Among the latter we should be inclined to place first the sections on poisoning, which are an excellent synopsis of that by no means easy subject. The spelling, it may be added, is American, and the prescriptions are apparently also based upon the U.S.A. Pharmacopœia.

### A Handbook of Practical Points.

Hygiene and Public Health. By Louis C. Parkes, M.D., D.P.H., and Henry R. Kenwood, F.R.S., D.P.H. Fifth Edition. Illustrated. (London: H. K. Lewis. 1913. Price 10s. 6d.)

There are a few books which, although coming under the category of text-books, can be justifiably described as particularly suited for institutional workers. They should undoubtedly find a place either in the library or in the secretary's office. The book at present under notice is distinctly one of these works. As the favourite text-book for public health students and medical officers of health, it is, of course, well known, and holds a well-assured position in its class. But perhaps its usefulness as a reference-book on a large number of subjects coming almost daily within the purview of workers in even a general hospital is not quite so fully realised as it should be. It is hardly necessary to describe in detail the features of what is now a standard text-book; but from the point of view of institutional officers we know of no other book from which practical information can be more conveniently gleaned on such subjects as ventilation, warming and lighting, hospital planning, disinfection, and sanitary law. This last section has always been well done, and in the present edition has been further extended by the inclusion of several new enactments of great practical importance, so that on this branch of knowledge alone the book forms a most serviceable compendium of the intricacies of public health law. The treatment of other matters remains well-up to date, and leaves no doubt in the mind that the reputation of the book is well deserved.

A Manual of Medical Treatment. By J. Burney Yeo, M.D., F.R.C.P. Fifth Edition. Edited by Raymond Crawfurd, M.D., F.R.C.P., and E. F. Buzzard, M.D., F.R.C.P. (London: Cassell and Co. 1913. 2 vols. Price 25s. net.)

This well-known manual of medical treatment has now reached a fifth edition and a size of 1,680 pages. It is an old favourite with the medical profession, and the work of revision has been entirely handed over by the original author to his two editors, Drs. Crawfurd and Buzzard, of whom the latter is responsible for the sections on nervous diseases alone and the former for all the rest. In spite of its many and conspicuous merits, not the least of which is the enormous store of practical clinical wisdom on which the author and his assistants can draw, we confess to a feeling of disappointment with the work as it now stands. For one thing, it is much too long-winded and verbose; we would undertake to cut it down by quite 400 pages without making any omission of real consequence; and even more drastic pruning still would do no very great harm. The style, in fact, is not sufficiently businesslike. Next, it is too indefinite in many respects; a couple of dozen different kinds of treatment for one condition, each of which "may" do good, are not at all infrequently set forth for adoption. Admittedly, such caution is often salutary, especially concerning new kinds of treatment which are still on trial; but, all the same, a less vague tone of either appreciation or denunciation might more often be adopted. Thirdly, in our judgment, there is a distinct undervaluation of operative measures, and too much of the nineteenth-century physician's view of surgery as a last resort when all else fails. Appendicitis and gall-stones are instances of this, though others could be cited to the same effect. Then, again, in the sections on various diseases a good deal of space is devoted to ætiology and diagnosis. The former is out of place, except in the barest outline, in a manual of treatment; the latter is, of course, useful and even necessary, but is in many instances not at all brought up to date. Duodenal ulcer is a case in point, if one is needed. These criticisms apply less to Dr. Buzzard's chapters than to the bulk of the work. Possibly they have been prompted by comparison of these volumes with treatises on the same subject which have been published within the last three or four years; certainly this can no longer be regarded as the leading monograph on treatment.

The Nauheim Treatment of Diseases of the Heart and Circulation. By Leslie T. Thorne, M.D., B.S. Fourth Edition. (London: Baillière, Tindall, and Cox. 1913. Illustrated. Pp. 104. Price 3s. 6d.)

The indiscriminate use of carbonated baths in England has caused much disappointment both to practitioners and to patients, and has brought wholly undeserved discredit upon the treatment. But these failures have been for the far greater part entirely due to ignorance on the part of the administrator, and not to the treatment. Dr. Thorne's little book, now in the fourth edition, has done and will do good service in dissipating some of this ignorance and, what is more important, assist in giving this treatment that place of importance as a therapeutic agent which it rightly deserves. Properly used, the results of this treatment in England are in no way less satisfactory than those obtained at Nauheim. This small work also contains a fully illustrated section on the Schott exercises, which are such an important adjunct to the treatment. The present edition has been revised and amplified, and the records of old cases have been brought up to date.

### The Way of Success.

How to Succeed as a Trained Nurse: Being a Guide to her Remunerative Employment, with Particulars of the Various Openings where the Services of Private Nurses are in Demand. By Sir Henry Burdett, K.C.B., K.C.V.O. (London: The Scientific Press, Ltd. Price 2s. 6d.)

We have read with pleasure and profit Sir Henry Burdett's admirable little volume "How to Succeed as a Trained Nurse." There can be no doubt that this carefully compiled book supplies a real need, and nurses who are contemplating their future, without any definite plan as to the branch of nursing which they desire to take up after completing their training, will be able to gather from this useful volume full information about every branch of the profession. Many nurses make up their mind that they would like to try some fresh branch of nursing, without having any idea of the special duties connected with it, or the remuneration attached to the appointments in question. In future they will only have to procure this well-arranged Guide to discover the main facts concerning any fresh position they may desire to take up, together with clear instructions as to where to apply for further information concerning the chance of vacancies. Matrons, who are so frequently applied to by the nurses they have trained for guidance as to their future, will be glad to have this source of varied and up-to-date information at hand. We have no doubt that the facts accumulated with such care will be kept up to date in the same way as the other standard works by the same author, which are so familiar to all hospital workers.

A careful perusal of the advice, so evidently given with practical knowledge and experience of the matter under consideration, should prevent those nurses who are apt to act impulsively from making many mistakes. We would specially call the attention of nurses to the definite and wise advice given to any nurses who may contemplate starting nursing homes. Indeed, it is evident that every subject dealt with has not only been studied by the writer, but that his comments are the result of exceptionally wide experience. The desire to help trained nurses by warning them of the difficulties they must be prepared to face, and setting before them the importance of aiming at the highest ideals, while carrying out their practical duties, will commend itself to all true nurses, and these will heartily endorse what Sir Henry Burdett has to say on page 8 as to "the right spirit to cultivate." We are not sure that we agree with the author's dictum that the difficult work of private nursing is undertaken too soon after the conclusion of training. Many doctors complain that if private nursing is taken up by nurses who have been too long in hospital work, their familiarity with hospital routine is apt to make them too much inclined to carry these unnecessary methods into private nursing, much to the detriment of the comfort of private patients. However, we quite agree with the writer that the difficult work of efficient private nursing needs special training, and it is certainly true that some women who make admirable private nurses are not very successful in hospital work, and that many good hospital nurses lack the adaptability and the personality which goes so far towards making a private nurse an unqualified success. It is a lamentable fact that private nurses who work on their own account for any length of time are apt to deteriorate in the standard of work and conduct which had become habitual to them when they lived in the atmosphere of hospital life. We cordially endorse the author's statement that the Private Nursing Associations which follow the pernicious custom of sending out the next nurse on the list, irrespective of her suitability for the case in question, adopt an unsatisfactory, mechanical method of business mismanagement which can never secure success.

We can only recommend nurses who wish to understand what opportunities are open to the nursing profession at the present day, in every branch of their work, to invest half-a-crown on "How to Succeed as a Trained Nurse," and to study the little volume carefully. It should certainly be in the libraries of all nurses' homes.

